# Filthy lucre: A metagenomic pilot study of microbes found on circulating currency in New York City

**DOI:** 10.1371/journal.pone.0175527

**Published:** 2017-04-06

**Authors:** Julia M. Maritz, Steven A. Sullivan, Robert J. Prill, Emre Aksoy, Paul Scheid, Jane M. Carlton

**Affiliations:** 1 Center for Genomics and Systems Biology, Department of Biology, New York University, New York, United States of America; 2 Accelerated Discovery Lab, IBM Almaden Research Center, San Jose, California, United States of America; National Cancer Institute, UNITED STATES

## Abstract

**Background:**

Paper currency by its very nature is frequently transferred from one person to another and represents an important medium for human contact with—and potential exchange of—microbes. In this pilot study, we swabbed circulating $1 bills obtained from a New York City bank in February (Winter) and June (Summer) 2013 and used shotgun metagenomic sequencing to profile the communities found on their surface. Using basic culture conditions, we also tested whether viable microbes could be recovered from bills.

**Results:**

Shotgun metagenomics identified eukaryotes as the most abundant sequences on money, followed by bacteria, viruses and archaea. Eukaryotic assemblages were dominated by human, other metazoan and fungal taxa. The currency investigated harbored a diverse microbial population that was dominated by human skin and oral commensals, including *Propionibacterium acnes*, *Staphylococcus epidermidis* and *Micrococcus luteus*. Other taxa detected not associated with humans included *Lactococcus lactis* and *Streptococcus thermophilus*, microbes typically associated with dairy production and fermentation. Culturing results indicated that viable microbes can be isolated from paper currency.

**Conclusions:**

We conducted the first metagenomic characterization of the surface of paper money in the United States, establishing a baseline for microbes found on $1 bills circulating in New York City. Our results suggest that money amalgamates DNA from sources inhabiting the human microbiome, food, and other environmental inputs, some of which can be recovered as viable organisms. These monetary communities may be maintained through contact with human skin, and DNA obtained from money may provide a record of human behavior and health. Understanding these microbial profiles is especially relevant to public health as money could potentially mediate interpersonal transfer of microbes.

## Introduction

Metagenomics has revolutionized the analysis of complex microbial communities by enabling the identification of unculturable microbes in environmental samples [1, 2]. Shotgun metagenomic studies of soil [3], sea [4], and a variety of human body sites [5] have provided insights into the diversity, life cycles, and function of many microbial communities, and their involvement in the health and well-being of humans, wildlife, and the environment. People encounter microbes on virtually every surface they touch and this daily contact has the potential to influence their well-being, yet we know very little about the microbial reservoir of urban surfaces or how they influence human health. Characterizing the microbial composition of surfaces is an important first step to understand the interactions between humans and microorganisms, but also serves as a means to monitor and potentially control the spread of diseases.

Recent research into the microbial aspect of human interaction with our surroundings has profoundly changed our understanding of how we impact environmental microbial communities. Each of us has a specific microbial “fingerprint” that is transmissible through exhalation or physical contact, potentially transferring millions of microbial cells per event [6]. Microbial communities within homes [7] and offices [8] are highly similar to those of their occupants, and surfaces frequently contacted by human hands such as keyboards [9], cell phones [10], ATM buttons [11], and subway holds [12] have microbial communities largely composed of skin taxa. Surface type, frequency of use, the identity of the interacting individuals, and biogeography also influence surface microbial communities [13–16].

Paper money offers an attractive window into human-driven microbial community diversity due to the high frequency of manual currency exchange in commerce, food service, the sex trade, and travel—activities that are likely to strongly influence the types of organisms present. The hygienic status of banknotes has been a topic of speculation since the late 1800s [17]. *In vitro* culture studies have established that paper currency can harbor high levels of microbes, some of which are clinically significant, such as the causative agents of pneumonia and enteric diseases [18–20], and various antibiotic resistant strains [21]. Laboratory simulations also provide evidence that many microorganisms can survive on office paper [22], coins [23] and banknotes of various materials [19, 24]. These studies, though limited, have established that microbial contamination of paper currency is widespread, and that money represents an important human-microbe interface.

We designed a series of pilot experiments to answer several questions regarding the diversity of microbial communities on circulating paper currency in New York City. In the United States, $1 bills have the highest volume [25], and the shortest average life span (5.8 years compared to 7.9 years for $20 bills and 15 years for the $100 bills [26]) of all circulating currency. As such, $1 bills can be considered a highly trafficked surface that experiences frequent human contact, and were chosen for this study on this basis. Using shotgun metagenomic sequencing, we characterized and compared the diversity found on $1 bills obtained from a bank in the heart of Manhattan, and tested whether viable microbes could be cultured from them *in vitro*. To our knowledge, this is the first direct exploration of the microbial composition of circulating paper money in the United States using high-throughput Illumina sequencing and metagenomic analysis techniques.

## Results

**Fig 1:**
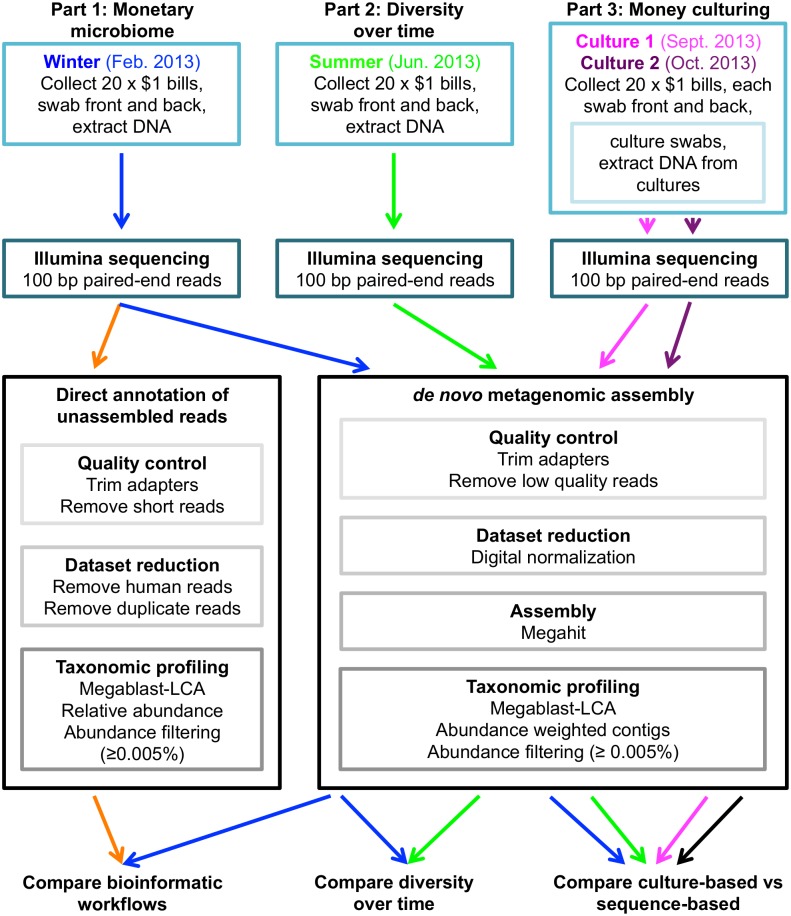
Flowchart describing the experimental plan and workflow for sample collection, sequencing and metagenomic data analysis.

We used a three-part approach to create a metagenomic profile of circulating paper currency (illustrated in Fig 1). In part one, we investigated whether microbial DNA was detectable on the surface of $1 bills and compared bioinformatic workflows for metagenomic analysis to determine which was optimal. In part two, we collected $1 bills from the same bank six months later to compare the monetary microbial communities over time. Finally, we determined whether viable microbes could be cultured *in vitro* from $1 bills and how those communities compared to the communities recovered using only sequence-based characterization methods.

### Metagenomic analysis of the monetary microbiome

We collected and swabbed twenty $1 bills from a bank in Manhattan in February 2013. DNA from these bills was pooled into one sample for library preparation and subjected to shotgun metagenomic sequencing, resulting in 544,298,464 reads. We analyzed the metagenomic data from this sample using two different methods: (1) direct annotation of unassembled reads and (2) *de novo* metagenomic assembly of reads using the Kalamazoo Metagenome Assembly Pipeline [27] (see Materials and methods, below).

**Fig 2:**
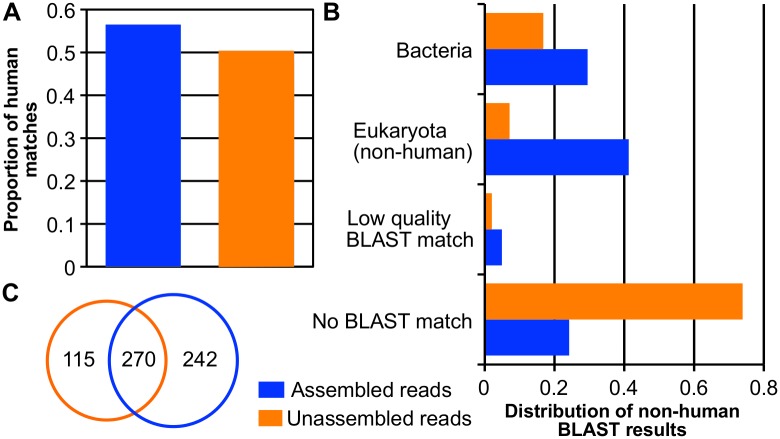
Comparison of organisms identified in the Winter 2013 data by direct annotation of unassembled reads and *de novo* metagenomic assembly analysis methods. **(**A**)** Proportion of human matches identified by Bowtie2 (unassembled reads) and megaBLAST-LCA (assembled reads). (B) Proportional distribution of non-human megaBLAST-LCA results. Low quality matches are defined as those filtered out prior to LCA analysis (see Materials and methods). High quality BLAST matches are broken down by taxonomic category and only categories with proportions >1% are included. (C) Number of bacterial identified in each dataset after abundance filtering to ≥ 0.005%.

Both methods detected a diverse monetary microbiome including prokaryotes, eukaryotes, archaea, and viruses. Human sequences were the most abundant, representing about half of the high-quality reads in each method (Fig 2A). Of the non-human reads nearly three quarters (73.9%) of the unassembled ones had no match in GenBank; bacterial reads were next most abundant (16.8%), followed by non-human eukaryotes (7.1%), viruses, and archaea (both < 1%) (Fig 2B). In the assembled data, eukaryotes represented the largest nonhuman category (41.2%), followed by unclassified reads (32%), bacteria (29%), viruses, and archaea (both again <1%) (Fig 2B). The assembled data identified far fewer bacterial taxa at the species level (2,115 unique species with at least one hit) than the unassembled data (7,580 unique species with at least one hit); however, the difference shrinks (385 vs. 512) after abundance filtering to ≥ 0.005% (~81% of total bacterial reads in each method), with 270 species (43.1%) overlapping (Fig 2C). As the *de novo* metagenomic assembly approach provides longer sequences for more robust classification, and read abundance in the contigs can be used to properly weight proportions, our subsequent analyses focused on results obtained with this method.

### Eukaryotic sequences dominate paper currency

**Fig 3:**
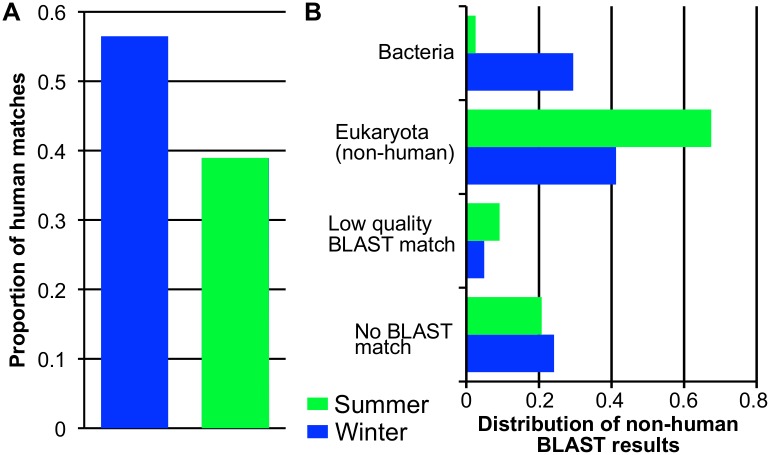
Differences in the abundance of organisms identified on $1 bills collected in Winter vs. Summer 2013. (A) Proportion of human matches identified by megaBLAST-LCA for each time point. (B) Proportional distribution of non-human megaBLAST-LCA results. Low quality matches are defined as those filtered out prior to LCA analysis (see Materials and methods). High quality BLAST matches are broken down by taxonomic category and only categories with proportions >1% are included.

To sample monetary microbiome diversity over time, we collected an additional set of twenty $1 bills from the same bank in June 2013. DNA from these bills was pooled into one sample for library preparation and subjected to shotgun metagenomic sequencing, generating 476,898,204 reads. Both the February and June 2013 read sets (referred to henceforth as Winter and Summer) contain matches to prokaryotes, eukaryotes, archaea, and viruses. In both sets the number of archaeal and viral sequences detected was low (< 1%), and were not analyzed further. Human sequences were the most abundant in the Winter data (56.4%), but were much less abundant in the Summer data (39%) (Fig 3A). At both time points eukaryotes represent the largest portion of non-human reads, albeit much smaller in Winter (41.2%) than Summer (67.4%; Fig 3B). Metazoan BLAST matches were the dominant group in Summer, with top hits to *Equus caballus* (horse, 49%), followed by *Sus scrofa* (wild boar, 6%), and *Canus lupis* (grey wolf, 2%). Although these are the top-ranking BLAST hits, *Sus scrofa* and *Canus lupis* are likely artifacts of Genbank and represent the presence of other eukaryotic genomes that are more prevalent in an urban environment (e.g. dogs and domestic pigs). *Equus caballus* was also the most abundant taxon in Winter (3.5%) followed two fungal species *Aspergillus niger* (1%) and *Wallemia sebi* (1%), both of which are commonly found in indoor environments, and associated with contaminated food [28–30]. For bacterial reads the opposite was true, with Winter bills yielding a higher proportion than Summer (Fig 3B).

### Money microbial communities are largely derived from human skin and oral commensals

A total of 397 bacterial species representing more than 20 bacterial phyla were identified across aggregate samples, though nearly all sequences (at least 97% of each bacterial dataset) belonged to just three phyla, the Actinobacteria, Firmicutes, and Proteobacteria, taxa that are representative of human skin communities and have previously been shown to dominate other urban surfaces, including the subways of Boston [12] and New York [31], and ATM keypads in New York [11]. As expected based on the distribution of the BLAST results (Fig 3B), many more bacterial taxa were present in Winter (385 species total) than in Summer (149 species total), with 137 found in both.

**Fig 4:**
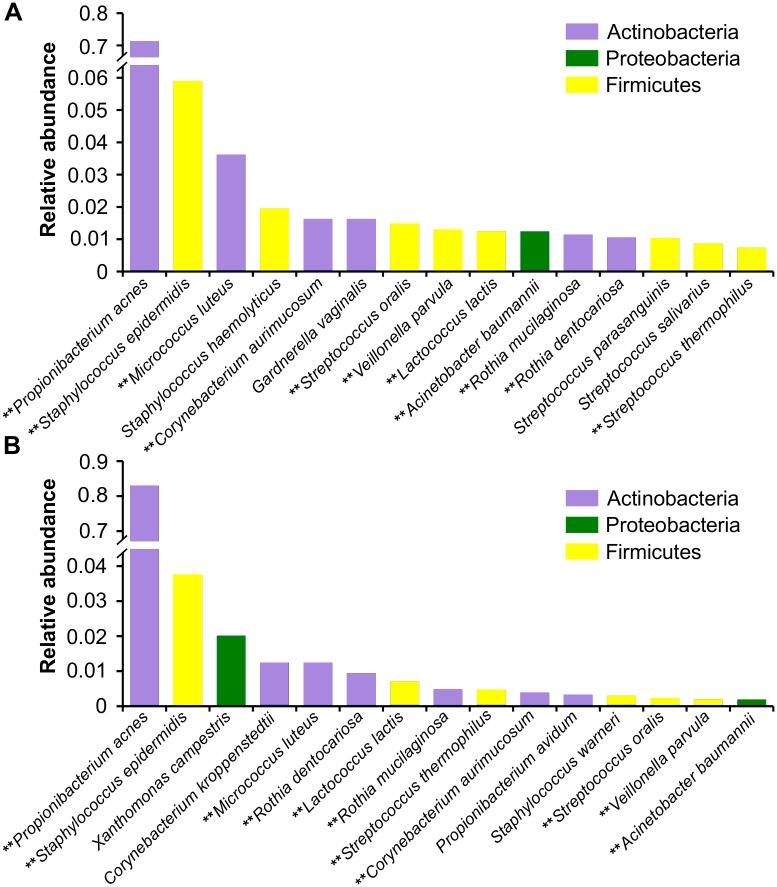
Relative abundance at the phylum and species level of bacterial taxa identified in the Winter and Summer 2013 datasets. Relative abundance of the top 15 bacterial taxa at the species level for (A) Winter and (B) Summer. The color of each species corresponds to its respective phylum. Those species marked with a ** indicate species present in the most abundant of both time points.

Similar bacteria dominated the two time points, including eleven of the most abundant bacterial species (Fig 4). The most abundant bacterial species in both datasets was *Propionibacterium acnes*, one of the most common among the skin microbial community; however, it was much more abundant in Summer (83%) than in Winter (51%). Other human-associated taxa among the most abundant species at both time points include a common skin bacterium, *Staphylococcus epidermis*; several oral taxa such as *Micrococcus luteus*, *Streptococcus oralis* and *Rothia* (*R*. *mucilaginosa*, *dentocariosa*) [32]; gut and oral commensal *Veillonella parvula* [33]; vaginally associated *Corynebacterium aurimucosum* [34]; and *Acinetobacter baumannii*, an opportunistic human pathogen [35]. Highly abundant taxa in both time points not directly associated with humans include *Lactococcus lactis* and *Streptococcus thermophilus*, microbes typically associated with dairy production and fermentation [36]. The majority of the other abundant bacteria at both time points are common skin and oral flora. Exceptions to this include *Gardnerella vaginalis*, found only in the most abundant taxa of Winter (Fig 4A), and *Xanthomonas campestris*, a bacterial plant pathogen that is also used to produce xanthan gum, found only in Summer [37] (Fig 4B).

### Viable microbes were isolated from money

**Fig 5:**
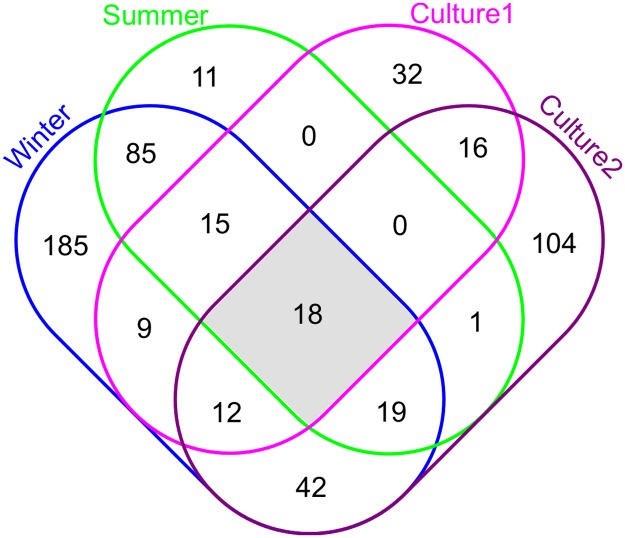
Number and overlap of bacterial species identified on money by the sequence-based experiments (Winter and Summer) vs. bacterial species cultured then sequenced from LB media (Culture 1 and Culture 2). Shaded area indicates species that were identified in all four experiments.

Microbial DNA found on paper currency is indicative but not sufficient evidence of live bacteria. To determine whether viable microbes could be isolated from money, we collected two additional sets of 20 $1 bills in September and October of 2013 (referred to henceforth as Culture 1 and Culture 2), swabbed the bills, plated the swab eluent on agar plates under sterile conditions, and incubated them at 37°C. Colonies appeared on all plates after 24 hours, and DNA was extracted and pooled into one sample per set of 20 bills after three days. We characterized the colonies by DNA sequencing and annotation, using the same quality control and assembly analysis methods described above (Fig 1). After abundance filtering we identified 102 bacterial species in Culture 1 and 212 species in Culture 2, far less than the Winter sequence-based characterization (385 species). The Summer sequence-based characterization identified more bacterial species (149) than Culture 1 but less than Culture 2, likely due to the significantly lower proportion of bacterial reads observed in the Summer dataset (Fig 3B). Of the 549 unique bacterial species identified in this study only 18 were observed in all four datasets, and only one, *Micrococcus luteus*, had a relative abundance above 1% (mean 2.3%) in all four experiments (Fig 5, S1 Table). Other taxa common to all four experiments include human-associated *Staphylococcus* (mean 3.95%), along with taxa not associated with humans, including soil taxon *Kocuria rhizophila* (mean 0.60%) (S1 Table).

We compared our monetary microbiome results to those of four previous culture-based studies from 1975–2009, which used morphological techniques to identify bacterial species. These studies sampled money of various denominations and composition, from the USA [18, 38], Nepal [39] and Egypt [40], and identified 20 different bacterial taxa, some of which are known to cause human infections (Table 1). Eighteen of the 20 bacteria taxa cultured in these studies were also found in our study, representing between 11–72% of the total bacterial reads for each time point. The majority of these taxa were identified at low levels in our dataset, particularly in our cultures where less than ten would have survived the abundance filtering step, but this could be due to the differences in culturing techniques, composition, and age of the bills used, or the use of sequence versus. morphological identification [41, 42].

**Table 1:** Comparison of bacteria identified in this study (proportional abundance of each species) to those cultured in four previous studies published during 1975–2009.

Species or group	Country	Bill type	Human Pathogen?	Winter	Summer	Culture 1	Culture 2	Refs
***Acinetobacter* species**	USA	Cotton	Some	**0.01741**	**0.00241**	**0.00070**	**0.00008**	[38]
*Alcaligenes* species	USA	Cotton	Some	0.00000	0.00000	0.00000	0.00000	[38]
***Bacillus* species**	USA	Cotton	Some	**0.00068**	**0.00006**	**0.19001**	**0.45776**	[38]
Diphtheroids*	USA	Cotton	No	**0.04176**	**0.02294**	**0.00013**	0.00036	[38]
*Enterobacter aerogenes*	Nepal	Polymer	Opportunistic	0.00005	0.00000	0.00000	0.00000	[39]
*Enterobacter cloaceae*	Nepal	Polymer	No	**0.00121**	0.00004	0.00004	0.00004	[39]
*Enterobacter* species	USA	Cotton	Some	**0.00140**	**0.00005**	0.00004	**0.00031**	[18, 38]
*Escherichia coli*	USA, Nepal	Cotton, polymer	Some	**0.00011**	0.00001	0.00000	0.00000	[18, 39]
*Escherichia vulneris*	USA	Cotton	Yes	0.00000	0.00000	0.00000	0.00000	[38]
*Klebsiella pneumoniae*	USA, Nepal, Egypt	Cotton, polymer	Yes	**0.00225**	**0.00010**	0.00000	0.00000	[38–40]
*Klebsiella* species	USA	Cotton	Most	**0.00367**	**0.00010**	0.00000	0.00001	[18, 38]
*Proteus mirabilis*	USA	Cotton	Yes	**0.00017**	**0.00032**	0.00000	0.00000	[18]
*Pseudomonas aeruginosa*	USA	Cotton	Yes	**0.00289**	**0.00013**	0.00000	**0.00301**	[18]
*Pseudomonas putida*	USA	Cotton	No	**0.00141**	**0.00022**	0.00000	**0.00097**	[38]
*Salmonella enterica*	Nepal	Polymer	Yes	**0.00006**	0.00001	0.00001	**0.00005**	[39]
***Staphylococcus aureus***	USA, Nepal, Egypt	Cotton, polymer	Yes	**0.00523**	**0.00148**	**0.02376**	**0.00077**	[38–40]
***Staphylococcus epidermis***	Nepal, Egypt	Cotton, polymer	No	**0.04827**	**0.03505**	**0.00893**	**0.00038**	[39, 40]
***Staphylococcus species***	USA	Cotton	Some	**0.08165**	**0.04249**	**0.50118**	**0.04339**	[18, 38]
*Streptococcus pyogenes*	Nepal	Polymer	Yes	**0.00066**	0.00001	0.00000	0.00000	[39]
*Streptococcus* α-hemolytic	USA	Cotton	Some	**0.05228**	**0.01303**	0.00000	0.00004	[38]
Total	NA	NA	NA	0.26116	0.11847	0.72482	0.50717	NA

Bold numbers indicate a proportional abundance ≥ 0.005%; bold taxa have ≥ 0.005% in all four datasets.

*Used to represent nonpathogenic *Cornynebacteria*.

## Discussion

Our work is the first metagenomic characterization of microbial diversity present on paper money circulating in New York City, the financial capital of the world. Our data indicate that paper money—the site of a potential “monetary microbiome”—harbors DNA from all four primary branches of the tree of life, and harbors living bacteria. As a whole, these results confirm and deepen several previous studies suggesting that money harbors a diverse and viable microbial population.

The majority of the sequences identified in our study were eukaryotic. We expected to detect high levels of human DNA on currency; however, we found much less than might be expected, particularly in the Summer bills. However, these bills also had a much higher proportion of non-human eukaryotic DNA, with high incidences of *E*. *caballus* (horse), *S*. *scrofa* (wild boar), and *C*. *lupis* (grey wolf). Although these are the top-ranking matches for these reads it is highly unlikely that wild boars and wolves are present in New York City. More likely, these are false positives generated by best-hit analysis and represent the presence of other mammalian species that are more prevalent in urban environments. This DNA may be left on bills through skin-mediated contact or environmental exposure during activities such as visiting parks, paying for meals or having pets at home. The higher occurrence of these species on money in Summer could reflect a seasonal increase in these activities, many of which take place outdoors during warmer months, while bills collected in Winter show higher levels of common indoor fungi.

Although the majority of sequences identified were eukaryotic, our $1 bill microbiome also included more than twenty bacterial phyla across aggregate samples, with the majority of sequences representing Actinobacteria, Firmicutes, and Proteobacteria, phyla that dominate the microbiomes of human skin [43] and oral cavities [44]. In agreement with previous studies, the most abundant taxon was *P*. *acnes*, which is commonly found on sebaceous (oily) areas of the skin, such as the face [45, 46]. The prevalence of human-associated taxa on paper money is unsurprising, as many previous studies have shown that the microbial communities of common surfaces closely resemble those of the human body parts that contact them [13, 47, 48]. We also observed high abundances of oral- and food-associated bacterial taxa on bills at both time points, further suggesting that actions performed before and during handling, such as face-touching, eating, or finger-licking prior to counting may influence community composition.

As no DNA was detected on new bills that we procured, or on the Winter and Summer bills re-swabbed six months later, paper money most likely acquires these communities during human-mediated exchange of bills. While our study design does not allow us to pinpoint the exact source of these communities, several studies have shown associations between human activities and the community compositions of skin and surfaces [49–53] and previous analysis of other NYC surfaces also detected “molecular echoes” [16] of human activities, particularly from recent meals, on both surfaces in the urban transit system [31] and on ATM keypads [11], suggesting that residual DNA/microbes may persist on a person’s hands and be transferred to surfaces upon use.

One goal of studying the microbiomics of highly trafficked surfaces such as money is to establish a baseline through which variability can be tracked and used for public health information. The bulk of bacteria found on the surface of money in this study were not associated with any human disease and the profiles were similar over time. However, we detected some taxa with potential implications for human health. Most were present at very low abundance, except *G*. *vaginalis*, which is associated with disruption of the vaginal flora/vaginosis, and *A*. *baumannii*, an opportunistic pathogen with a high incidence of multidrug resistant strains [35]. These taxa were more abundant in the Winter bills than the Summer ones, which could reflect biogeographic and/or seasonal patterns in the health of the individuals who handled the bills, highlighting the potential use of money as a fomite to map public health trends.

Detection of microbial genetic material via sequencing is not sufficient to demonstrate viable microbes, so we undertook a basic culturing experiment and determined that viable microbes can be recovered from paper currency. Our sequence-based experiments generally identified a greater diversity of bacterial species than the culture-based experiments, probably because DNA sequencing techniques can detect organisms that cannot be cultured; the culture results only represent those bacteria capable of growing on LB-agar plates at 37°C, and the different environmental conditions at the time of each collection could influence the type and amount of microbes present on the bills.

The present study aimed solely to characterize the microbial diversity found on $1 bills, and we had no way of assessing “transitory” vs. “resident” taxa aside from the obvious metazoan species. Although we detected DNA signatures similar to bacteria with pathogenic potential, this does not provide any definitive information regarding their pathogenicity, and sequence-based data by itself does not indicate that these reads were from live pathogens. Previous metagenomic studies have also identified organisms with pathogenic potential on some urban surfaces [12, 31, 54]; however outright virulence factors were rare, indicating that the pathogenic potential of these organisms is very low.

Furthermore, our detection of viable microbes on paper currency does not establish their source, nor does it affirm that they can be stably transferred from one individual to another. Preliminary studies have demonstrated the potential of money to facilitate the transmission of microbes and act as a disease vector, particularly in the presence of food [55, 56]. However, in-depth complementary laboratory investigations are required are to determine if the microbes found on money are deposited through human contact, whether they are metabolically active or dormant, their survival time, whether they can be stably transferred from money to individuals, and the potential for infection.

Our study provides only a glimpse of the NYC monetary microbiome, in terms of sample size and number of time points, and greater diversity is likely present. Establishing a “typical” microbial profile for money will require further large-scale studies with greater longitudinal depth, covering multiple locations, sources (including stores, food vendors, hospitals, etc.) and denominations of bills. Nonetheless the present dataset helps to characterize the “urban microbiome” of a major city, and in doing so provides a jumping off point for future hypothesis testing, hinting at potential large-scale trends that may influence the distribution and persistence of microbes on paper currency and money as a potential human-microbe interface. The nature of these communities and their potential transmission during hand-to-hand exchange will be important areas of future research, as microbes that can be transmitted between people via money could have considerable health implications and their identification has the potential to inform public health and policy.

## Materials and methods

### Sample collection, processing and sequencing

Twenty $1 notes were collected from a bank in Greenwich Village, New York City in February 2013, representing the winter sample, and brought back to NYU for processing. A sterile ESwab (Copan Diagnostics), moistened in molecular biology grade water (Fischer Scientific), was used to swab the front and back of two individual bills, and repeated until all bills were swabbed (10 swabs total). DNA was extracted from each swab with the PowerSoil DNA Isolation kit (MOBIO, catalog #12888) with the following modifications: (1) moist swabs were cut approximately 0.25 inches from the tip end and placed directly into the supplied bead tube; and (2) residual liquid from the swab tip was extracted post bead-vortexing using filter-less spin-columns (Qiagen). DNA from the ten swabs was concentrated post-extraction using a SpeedVac (Savant) and subsequently pooled for optimal biomass recovery. The same process was repeated again in June 2013 on an additional 20 $1 bills, representing the summer sample. This resulted in two samples, winter and summer, each representing DNA from 20 $1 bills.

Two additional groups of twenty $1 notes were collected from the same bank in September 2013 (Culture 1) and October 2013 (Culture 2) and swabbed as described above. Swabs from each collection were deposited together into Falcon tubes containing 5 ml of LB (Luria Broth). After vigorous mixing, 1 ml of LB was spread onto five different LB agar plates per collection, and the plates incubated at 37°C for 72 hours under aerobic conditions. Colony growth was monitored every 24 hours, and after 72 hours colonies from each plate were scrapped from the media surface and used for DNA was extraction. One DNA extraction was performed per plate and DNA from each set of 20 bills was pooled into one sample as described above. This resulted in four samples total, each representing DNA from 20 $1 bills, two from swabbed bills (winter and summer collections), and two from cultured bills (fall collections).

Several controls were included to ensure a contaminant-free environment. First, all swabbing and culturing of all bills was performed under Bio Safety Level 2 conditions in a laminar flow hood, with swabs of the hood and ambient air included as negative controls. No measurable quantities of DNA were extracted from unused moist swabs, swabs of the laminar flow hood surface or swabs exposed to ambient air where extraction occurred, and no colonies were observed on control plates cultured from these swabs, indicating that the harvested genetic material in our study was not attributable to the working area, laboratory equipment or ambient air. In addition, swabbed bills from February 2013, and June 2013, which had been stored in an airtight container, were re-swabbed, and twenty new $1 bills that had yet to be circulated through New York City were also collected and swabbed. DNA was not recovered from re-swabbed or fresh bills, and these samples were discarded from further sequencing and analysis.

Metagenomic libraries were constructed from pooled DNA using the Illumina Library Preparation kit (Kapa Biosystems) following the manufacturer’s instructions with a modification to increase the frequency of ligated fragments. Adapters (Bio Scientific) were ligated for 75 min at 20°C then subjected to a cycling gradient (10–30°C every 30 sec) [57]. Purified libraries were size selected in the range 200-900bp using a Pippin Prep (Sage Science) and sequenced on the Illumina HiSeq2000 platform with 2x100 bp paired-end chemistry. The winter and summer libraries were sequenced on individual HiSeq lanes, while the two libraries from cultured bills were pooled and sequenced on one lane of a HiSeq Rapid Run.

### Metagenomic data analysis

#### Direct annotation of unassembled reads

Raw sequence data were: (1) demultiplexed using CASAVA (Illumina), (2) trimmed of adapter sequences, and filtered of reads with uncalled bases and reads < 90 nucleotides in length using Flexbar [58]. Host DNA, or human reads presumably from individuals that handled the money, were identified by aligning against the human genome (GenBank build hg19) with Bowtie2 [59] and removed from the analysis. Duplicate reads were identified based on 100% sequence similarity and removed.

#### *De novo* metagenomic assembly method

Assembly based analysis of metagenomic reads was undertaken following the Kalamazoo Metagenome Assembly Pipeline, version 0.8.5 as described in [27, 60], with a few modifications. Briefly, demultiplexed data were trimmed of adapters using Trimmomatic [61] and quality filtered to a minimum Phred score of 30 using the Fastx Toolkit. Quality filtered reads were digitally normalized [62] using the default parameters (K = 20, coverage = 20 and K = 20, coverage = 5) and assembled using MEGAHIT, version 0.1.2 using the default parameters [63]. See S2 Table for summary statistics of each metagenomic assembly. Read abundance values for this assembled dataset were calculated by mapping all quality filtered reads back to the final contigs using the default parameters in Bowtie and the make-coverage.py script in the Kalamazoo Metagenome Assembly Pipeline.

### Taxonomic binning analysis using Megablast-LCA

The final sets of unassembled reads and assembled contigs were searched against the NCBI nucleotide (NT) database using the Megablast algorithm [64] and default parameters (E-value of 10). Hits were required to have a minimum length of 65 bp (of a 100 bp read), a minimum bitscore of 60, and to have a bitscore within ten percent of the highest bitscore for the read. Identifiers for high quality hits (GI numbers) were mapped to NCBI Taxonomy identifiers. To resolve the ambiguity arising from multiple hits to different taxa, each read was assigned to the lowest common ancestor (LCA) node in the NCBI Taxonomy tree that encompassed the set of hits for the read. For example, hits to multiple species of the same genus are assigned to the common genus by the LCA algorithm established by MEGAN [65]. Relative abundance estimates were calculated for different categories as proportions of input data for the unassembled reads, and the contig read abundance values calculated above were used to properly weight abundance estimates for the assembled dataset. For robust analysis only species with a relative abundance of ≥ 0.005% were used for taxonomic profiling.

## Supporting information

S1 Table Proportion of abundance filtered reads for the bacterial species observed in all four experiments conducted in this study. (XLSX)

S2 Table Per-sample summary statistics of the metagenomic assembly. (XLSX)
